# Assessment of sapropel use for pharmaceutical products according to legislation, pollution parameters, and concentration of biologically active substances

**DOI:** 10.1038/s41598-020-78498-6

**Published:** 2020-12-09

**Authors:** Ilona Pavlovska, Aneka Klavina, Agris Auce, Ivars Vanadzins, Alise Silova, Laura Komarovska, Baiba Silamikele, Linda Dobkevica, Linda Paegle

**Affiliations:** 1 Institute of Occupation Safety and Environmental Health, Riga Stradins University, Riga, Latvia; 2 Institute of Biology, University of Latvia, Riga, Latvia; 3 Faculty of Geography and Earth Sciences, University of Latvia, Riga, Latvia

**Keywords:** Biochemistry, Microbiology, Natural variation in plants, Plant development, Plant ecology, Plant evolution, Plant molecular biology, Disease prevention, Public health

## Abstract

Development trends need the necessity for wider use of the local resources and available natural materials are one of the priorities around the world. Freshwater sapropel is a common material in the water basement of the lakes, but still not sufficiently explored. The main goal of the project to start detailed and systematic research on the medical properties of sapropel to be obtained in Latvia, promote its scientifically based use in balneology, develop new medical procedures and services, and promote development of new exportable products. The results include the survey, sampling depths, and processing, evaluation of external signs, physical, chemical, and biochemical parameters, and evaluation of microbiological indicators. Active components from the sapropel samples extracted using the alkaline method. Sapropel extracts were characterized by organic carbon content, humic and fulvic acid concentrations, total phenolic content, trace metal and pesticide concentrations, total antioxidant status, and microbiological flora. Summarizing the article's main findings it was concluded that Latvian freshwater sapropel can be used as raw material for obtaining sapropel extract and use it in the preparation of pharmaceuticals and promote the development of new exportable products and services.

## Introduction

*Medical importance.* Sapropel might seem something mysterious and incomprehensible that can be found somewhere deep in the water and is sunlight inaccessible. However, an extremely interesting and useful material has long been a major success for health improvement and treatment. Sapropel used in medicine for a long time and is widely used in various health sectors, but still not sufficiently explored. Sapropel is a common material in the water basement of the lakes in Latvia^1–6^.

Sapropel is sludge sediment in lakes, with a fine structure that contains incompletely divided organic matter and microscopic aquatic life forms residues with trace of sand, clay, calcium carbonate, and other rock impurities^2,7–10^.

Sapropel is a pasty mass of light grey, pink, brown, brownish olive or almost black. Sapropel’s deposits in swamps and lakes only occurred on post-ice age, which took place in the Baltic States 12–15 thousand years ago.

Medical mud formed by complex biological transformations of Holocene sediments. The composition of the therapeutic mud depends on the location of the acquisition—freshwater, saltwater or thermal springs. Sapropel sludge is classified as inorganic sediment sludge, river or lake mud, organic sediment sludge, freshwater and saltwater lake mud, peat sludge, mixed sludge, volcanic sludge and artificial sludge^10,11^.

In ancient times, people considered that sapropel can cure almost any disorder, even improve the long-term effects on the skin. Even today it is attributed that sapropel is marvellous material for wide range applications.

Sapropel is a multifunctional and widely used medical treatment, and believed to be useful for lymphatic and circulatory enhancement, vascular strengthening, skin structure, cellulite and subcutaneous fat reduction. It has a pronounced antibacterial effect and enriches the body with calcium, magnesium, bromine, iodine, potassium, and amino acids. Sapropel has an antioxidant effect that improves skin structure, smoothens wrinkles and prevents new wrinkles, removes swelling, strengthens nails and hair, normalizes sebaceous gland secretion, helps hair loss. The therapeutic effect of sapropel helps to restore immunity, maintain the cellular structure of various skin diseases—dermatitis, seborrhea, acne, and other rashes and other skin diseases. However, today, sapropel preparations are most widely used in balneotherapy and cosmetology, especially in the treatment of chronic or protracted diseases^3,12–20^.

Sapropel complex chemical and biological structure explains its multifunctional effect on body. The bioactivity of sapropel determines by its humic acids, fulvic acids and heratomelic acids, various vitamins and microorganisms that release antibiotics. Previously, sapropel commonly used in raw form and there is no standard methods for sapropel extraction, generally. Currently, there are few extraction methods for getting bioactive components from raw sapropel^19^. Latvian freshwater sapropel could be used as raw material for getting sapropel extract and use it as remedy. All mentioned above brings us to the main question for sapropel usage in medicine, balneology and pharmacy “how to develop quality criteria for raw sapropel and its extracts”. The quality criteria should include minimum requirements for pollution levels (heavy metals, pesticides), biologically active substance concentration, pH values, antioxidants as well as physical characteristics^21,22^.

*Sapropel legislation.* It is important to monitor and inspect sapropel extraction sites to assess the level of contamination and the environmental impact of anthropogenic activity. Sediment contamination is considered to be a major environmental issue because sediment acts as a reservoir for pollution. Sediments are an integral part of the aquatic ecosystem, which provides food and habitat for various aquatic species.

Production of sapropel in the industrial scale in Latvia is regulated by several Laws and Cabinet Regulations. One of them is the *Environmental Protection Law*^23^, which is the main normative act in the field of environmental protection. The purpose of the law is to ensure the preservation of the quality of the environment and the sustainable use of natural resources.

The *Law on Environmental Impact Assessment*^24^ defines the activities that require environmental impact assessment. The need for an environmental impact assessment procedure for the extraction of sapropel in lakes is governed by Chapter IV Section I point 1 and point 25 of Annex 1.

Obtaining Sapropel must also comply with the *On Pollution Law*^25^. *Pollution*, purpose is to prevent or reduce damage to human health, property and the environment caused by pollution. The law sets out the procedures and guidelines that must be taken into account when performing polluting activities to minimize the impact on natural resources such as soil, air, and water. The planned extraction of minerals should take into account the emissions of water, and air pollutants.

*Law On the Conservation of Species and Biotopes*^26^ regulates issues related to the protection of protected species and habitats. One of the main aims of the law is to ensure biodiversity by preserving the fauna, flora, and biotopes characteristic of Latvia. Extraction of the sapropel can also pose a threat to the habitat in the lake and affect species diversity.

The acquisition of Sapropel must comply with the *Spatial Development Planning Law*^27^. When planning the extraction of mineral resources, the conformity of the intended land use with the municipal spatial plans shall be taken into account.

It is important to consider the *Protection Zone Law*^28^ when planning the acquisition of a sapropel. The main tasks of this Law are to determine the types and functions of protection zones. The task of certain areas shall be to protect different types of objects (natural, as well as artificial) from undesirable external effects, to ensure the exploitation and safety thereof or to protect the environment and people from the harmful effect of an object.

The purpose of the *Natural Resources Tax Law*^29^ is to limit the mismanagement of natural resources and environmental pollution, as well as to promote the introduction of new and improved technology that reduces environmental pollution.

When extracting mineral resources, the legal requirements regarding the management of hazardous waste and municipal waste generated by the equipment used in the extraction process *Waste Management Law*^30^ must be observed.

When obtaining sapropel on the industrial scale, it is also important to comply with a number of Cabinet Regulations, including the *Regulations on Lists of Specially Protected and Restricted Species*, No. 396^31^; *Regulations on List of Species of Specially Protected Habitats*, No. 350^32^; *Rules on the criteria used to assess the significance of the impact of damage to particularly protected species or habitats*, No. 213^33^.

With regard to the extraction of sapropel, it is necessary to assess its impact on lake water quality; to ensure that the environmental quality standards for priority and hazardous substances in surface waters and the priority substances in the lake biota are not exceeded during the extraction process^34^.

*Regulations on the Discharge of Pollutants into Water*, No. 34^35^ establishes limit values and a prohibition for the emission of pollutants into water, as well as the procedures by which the operator controls the number of pollutants discharged into water, perform monitoring and provides relevant information.

Before starting the extraction of the sapropel, attention should be paid to the *Law on Subterranean Depths*^36^, which is one of the most important normative acts regulating the extraction of natural resources. It defines the procedure for the complex, rational and environmentally friendly use of subterranean depths. Pursuant to section 15 of that law in accordance with the procedures specified by the main requirements for the protection of subterranean depths which may be attributed to the extraction of a sapropel are the rational extraction of minerals and the use of by-products from the field; and use of subterranean depths without adverse effects on mineral resources and subterranean properties.

## Materials and methods

An important step is to determine the potential sources of sapropel accumulating within the depositional setting. Another consideration for site selection is to ensure that access granted from the relevant landowner and permission sought from the relevant agency if the site is designated as protected. It is vital to ensure that there is no risk of damaging any subsurface utilities (gas, electricity etc.)^19^.

**Figure 1:**
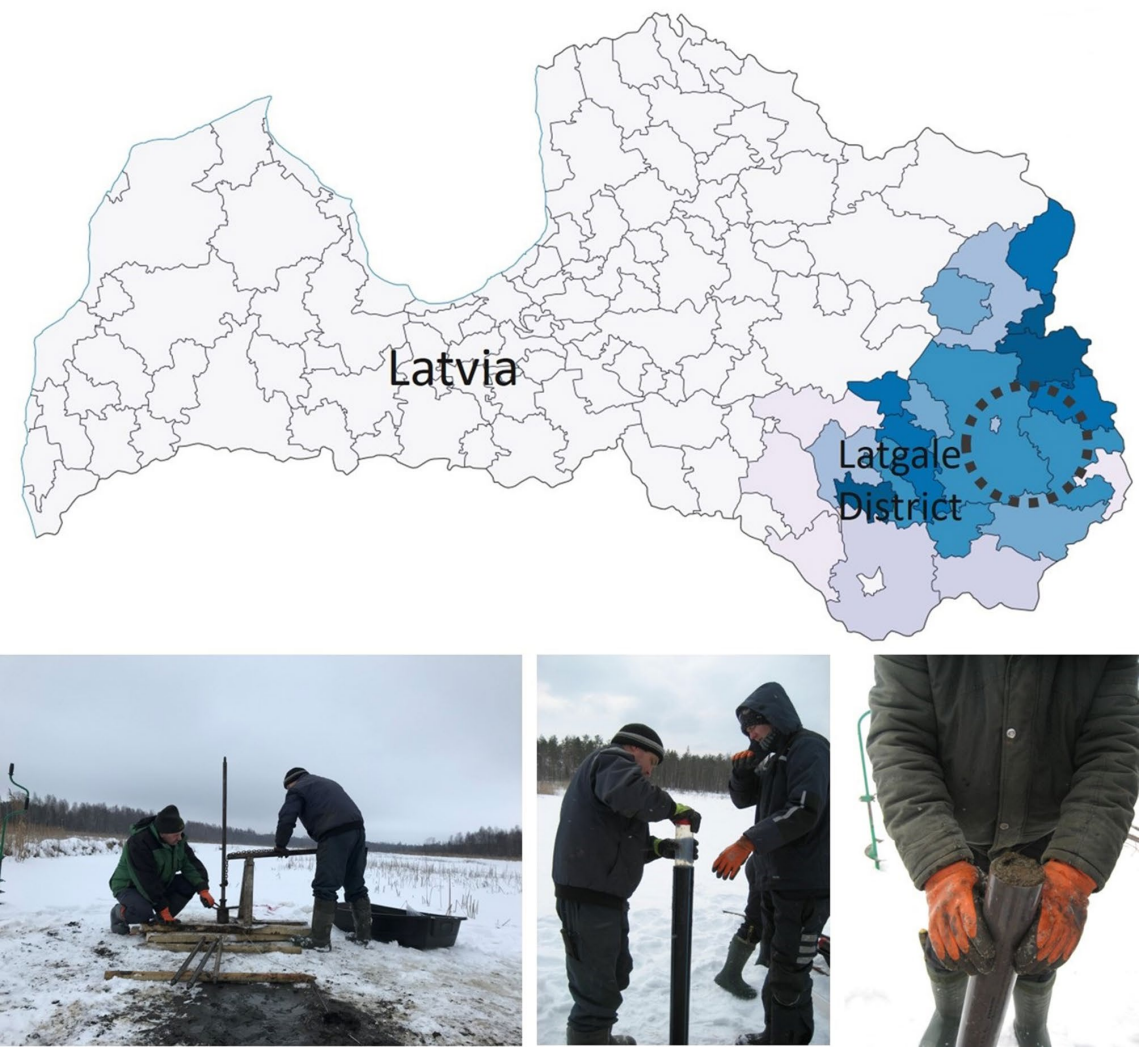
Sapropel extraction in Latgale District. Workers from geological research company “Geo Consultants, Ltd.” extract the samples. All pictures are the property of RSU Laboratory of Hygiene and Environmental Health, prepared in accordance with the *LVS EN 1997-2 AC:2014 L* (*Eurocode 7—Geotechnical design—Part 2: Ground investigation and testing*).

In most of the Latvian lakes, there is sapropel, in many swamps and under the peat layers, it also found. Major stocks of this mineral resource are around 300 million cubic meters, mostly located in Latgale districts, eastern Latvia (the light blue to dark blue region on a map, Fig. 1). Samples of sapropel were extracted from five lakes: Audzelu Lake (Istra rural territory), Dunakla Lake (Zvirgzdene rural territory), Ivusku Lake (Cornajas rural territory), Zeilu Lake (Cirmas rural territory) and Mazais Kivdalova Lake (Purenu rural territory). The location of lakes is marked with a circle in Fig. 1.

Official geological survey of Latvia lakes from Latvian lake database of association "Latvijas ezeri" (www.ezeri.lv) used in the selection of the area of the exploration.

The main selection criteria were the sapropel deposits depth, hydrological regime, the history of agriculture next to the lake and the potential exposure to industrial waste. One hundred and five sapropel samples obtained from five lakes (Zielu – Z, Mazais Kivdalova – K, Dunakla – D, Ivusku – I and Audzelu – A) during the wintertime (extraction equipment is installed on a platform over the ice and was therefore more stable).

Since the sapropel accumulates in the lake, there may be differences, depending on inflowing brooks and trenches in the lake, which may bring pollutants that are deposited closer to estuaries and also on the age/depth of the sapropel layer (therefore, there can be differences in the concentration of potential pollutants).

**Figure 2:**
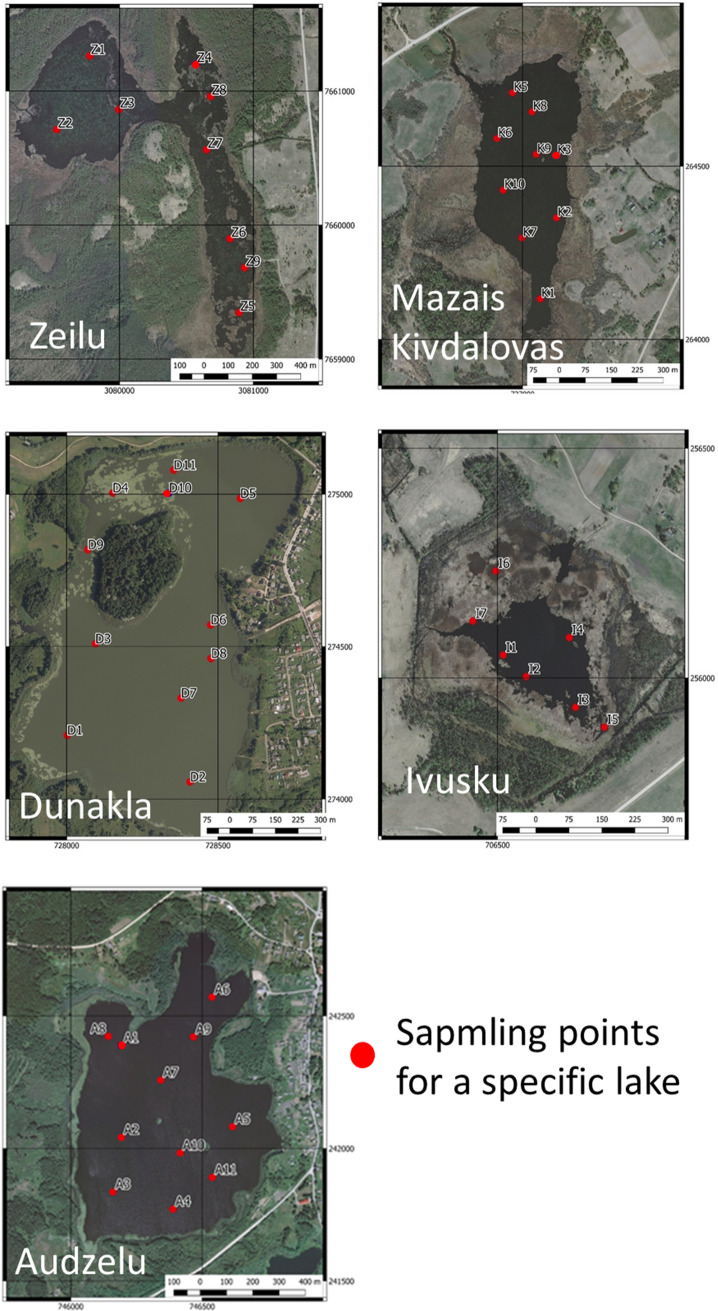
Overview of sapropel sampling points for a specific lake. All pictures were made in cooperation with geological research company “Geo Consultants, Ltd.” and are the property of RSU Laboratory of Hygiene and Environmental Health, prepared in accordance with the *LVS EN 1997–2 AC:2014 L* (*Eurocode 7 – Geotechnical design – Part 2: Ground investigation and testing*).

Prior to the sample collection, the thickness of the proper sediment layer was determined and the depth of sapropel deposit established for each of the lakes as well as within each of the lakes by taking probes. Well-composed sapropel layer for further laboratory analyses taken on the three different depths of sapropel sediment at each extraction point through the lake coordinates (Fig. 2). The amount of extraction points through the lake were different from 1 to 11 taking into account the lake dimension (Z 1–9, K 1–10, D 1–11, I 1–7 and A 1–11).

The lakes influenced by several external and internal factors. The soil conditions, climatic conditions, and access facilities to the main road and to the fields applied to mainly natural external factors. Internal factors depend on the type of business enterprises around, farmstead and the relative position of its different buildings. Among general principles that must be taken into account is the availability of transportation between buildings and driveway to the lakes.

To better explore the possibilities of using Latvian sapropel, Riga Stradins university (RSU) researchers have launched a three-year study to test and standardize a composition, properties, storage options and therapeutic effects of the sapropel.

There are main characteristics of the sapropel samples. Organoleptic properties – the color ranges from pale yellow to black, depending on the type of sapropel and the site of exploration. The texture is determined based on the initial description of the site. The smell is neutral (if any changes in the smell observed, the storage conditions of the samples should be checked). Sapropel must be homogeneous inconsistency, with no inclusions or excess water. Another one characteristic of sapropel is dry matter content. The sapropel is dried and weight loss compared to samples of a recognized sapropel site. This is mainly to determine if the series of raw materials are not obtained too shallow at the top of the sapropel layer. Sapropel is divided into four main types – organic, silica-containing, carbonate and mixed type sapropel. There are several kinds (peat, carbonated, iron-rich, mixed, silicate with increased ashes contain, etc.) of each type of sapropel, the main type being determined by the biological and oxide content of the sapropel^37^.

Sample collection carried out with a semi-cylindrical chamber with cone cap and longitude closed shutter made of stainless steel used with sample chamber dimensions 1000 × 75 mm. Samples from three different depths at the seven different localizations (21 samples in total) were established for each of the lakes.

The appropriate sapropel layer found from 2.0 to 9.0 m (experimental sapropel layer was from 0.9 to 11.4 m) from the surface of the sediment layer exact depth depending on the lake and the position of the measurement point. Actual thickness and location varied depending on the depth of the lake and degree of the decomposition of organic matter. If the depth is less than 1.5 m from the surface of the sediment layer, sapropel sediments are not fully developed and not used in this study.

**Figure 3:**
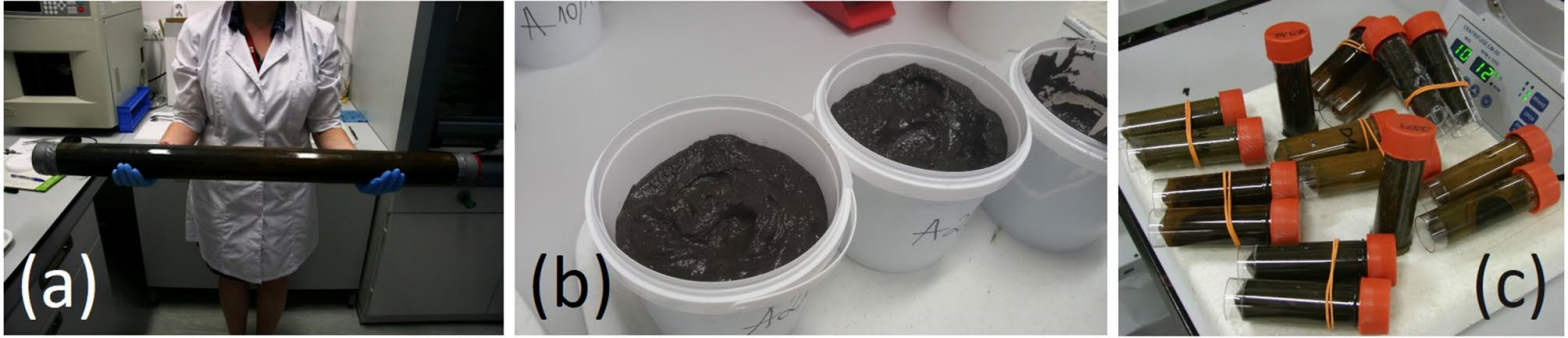
Storage of sapropel in the: (**a**) closed plastic containers, (**b**) removed from plastic containers and (**c**) selected samples for the extraction of active components.

Each sample identified by specifying the exact location of the site in the lake and the depth of extraction from the surface of the water and the beginning of the sludge layer. The sediments (Fig. 3a) were removed from plastic containers (Fig. 3b), then refrigerated, and kept at 4 °C (temperature closer to the natural water temperature at the bottom of the lake) and then, stored.

For the extraction of active components from the sapropel samples the alkaline method was selected (Fig. 3c)^38^.

Sapropel extracts were characterized by total organic carbon content (TOC), humic acid (HA) and fulvic acid (FA) concentrations by the use of the spectrometric method.

The sample pH level was determined using distilled water (volumetric ration sample: water – 1:2.5).

Sapropel samples were analysed for organic matter and carbonate content using the loss-on-ignition (LOI) method Loss on ignition. The dried sapropel sample was heated for 4 h at 550 °C and 2 h at 900 °C, after each heating the sample weighed and calculated by assuming that all organic matter in the sample is burned at 550 °C, and at the next temperature (900 °C). An important parameter of sapropel is the amount of organic matter. It can be determined whether organic substances mineralize by releasing their nutrients or accumulate in sediment and their mineralization process is slow. The amount of organic matter in the sediments of the lake can vary (from 20 to 90%), depending on the productivity of the lake and the type of land use in the catchment area. The carbonate content (from 1 to 15%), in turn, depends on the amount of carbonate soils in the catchment area, as well as on benthic organisms in molluscs whose shell may contain carbonates^39^.

Trace metal concentrations were determined in sapropel samples by electrothermal atomic absorption spectrometry with Zeeman background correction. Before starting the analysis, sapropel samples were dried at 105 °C and finely ground with a mortar and pestle. Sampling was carried out in a closed container with microwaves in the digested system using nitric acid and hydrogen peroxide. The contents of the vessels were quantitatively transferred to 20 mL graduated polypropylene flasks and filled to mark with Milli-Q water^38^.

The content of the total phenolic content (TPC) of the extract was expressed as gallic acid (GA) equivalents. The gallic acid was used to set up a standard curve. An aliquot of 500 µl of an extract was mixed with 2.5 ml of Folin-Ciocalteu phenol reagent (10 × dilutions) and allowed to react for 5 min. Then 2 ml of 7.5% Na_2_CO_3_ solution was added and allowed to stand for 1 h before the absorbance of the reaction mixture was read at 765 nm. All tests were performed six times. The total polyphenol contents of the extract were evaluated from the gallic acid standard curve and expressed as mg of gallic acid per gram of plant material^40,41^.

Total antioxidant status (TAS) in samples was measured using Randox Total Antioxidant status kit (Randox Laboratories Ltd.) adapted to the RX Daytona automated chemistry analyzer (Randox Laboratories Ltd)^41^.

2,2-diphenyl-1-picrylhydrazyl (DPPH) is a stable organic radical; in a chemical reaction, it functions as a radical and it is a scavenger of antioxidants. DPPH solution is violet with maximum absorption at 515 nm, while its reduced form is yellow. Therefore, the decreased level of absorption at 515 nm adding extracts was proportional to the natural substance antioxidant activity^41^.

The antiradical activity (six replicates per treatment) was expressed as IC50 (mg·mL^−1^)—the concentration required to cause a 50% DPPH inhibition. The ability to scavenge the DPPH radical was calculated by using the following equation: %_(inhibition)_ = 100·(A_0_ − A_1_)/A_0_, where A_0_—average absorption for the “empty” sample (contains solvent), A_1_—average absorption for the test sample^41^.

The calibration curve was obtained with TROLOX/methanol. The free radical scavenging activity for the sample was calculated after the Trolox equivalent and expressed in millimoles of Trolox equivalent (TE mmol·L-1) of the sample solution^41^. All antioxidant parameters were measured to sapropel extract with fulvic acid concentration 700 mg/L.

Detection and quantitation of dichlorodiphenyldichloroethylene (DDE)/dichlorodiphenyltrichloroethane (DDT) were realized by applying DDE/DDT ELISA kit.

Microbiological measurements were provided by the Institute of Food safety, Animal Health and Environment "BIOR". Test methodology for specific organisms reported the results in CFU (colony-forming unit), the actual count from the surface of a plate, applied the standard ISO 4833-1:2013 Part 1: Colony count at 30 °C by the pour plate technique^41,42^. This standard was last reviewed and confirmed in 2019, therefore the version remains current.

## Results and discussion

The main goal of the project is to carry out detailed and systematic research on the origin of sapropel to be obtained in Latvia and its possibility to use it for medical purposes, to promote its scientifically based use in balneology, to develop new medical procedures and services, and to promote the development of new exportable products in the nearest future. So far, studies have been basically focused on sapropel for other purposes, such as agriculture or cosmetology, thus without using sapropel and its acquired mud biomedical and biopharmaceutical potential.

Sapropel is a jelly-like homogeneous mass, its texture in upper layers is close to cream-like, and in the lower layers, it becomes denser. The sediments are odorless except for separate types that smell of hydrogen sulfide (Table 1). Sapropel color depends on organic substance and mineral additions and it refers to caustobiolites^43^. The temperature of 4 °C without exposure to light and oxygen were sufficient for preserving sapropel. Samples from different lakes and depths have different organoleptic characteristic and must be checked on color, texture, visual consistency, impurities and uniformity, as well as smell. Organoleptically sapropel samples found from greenish-yellow to almost black (Table 2).

**Table 1:** Description of the lakes.

	Zeilu	Mazais Kivdalova	Ivusku	Dunakla	Audzelu
Average depth of lake water layer (m)	1.1	1.3	1.0	2.2	2.0
Sapropel layer depth (m)	4.0–9.5	1.7–11.2	2.2–10.4	0.9–9.5	2.65–11.4
Lake surface area (h)	44.8	14.7	1.9	82.7	64.9
Lake bottom structure	Muddy	Muddy	Muddy	Muddy Gravelly	Muddy Sandy Rocky
Proximity of access roads (km)	0.5	0.2	0.2	0.5	0.2
Surroundings	Surrounded by reed beds, marshy forest; rural houses and service buildings; farmland; cemetery	Rural village Nuksi; rural houses with the adjacent area; field; forest	Farmland; cemetery	Close to town Ludza; one island in the lake	Populated area Vecsloboda; forest
Hydrological regime	Streams: 2 inflowing brooks and 6 ditches, 1 outflowing brook	Streams: 1 inflowing river and 3 ditches	Streams	Streams: 1 inflowing brook and 2 ditches, 1 outflowing river	Streams: 1 inflowing river, 1 brook and several ditches, 1 outflowing brook,
Sedimentological description of a section	Brownish grey to dark brown, flowing in upper layers, getting jelly-like in the lower layers, with plant residues, some H_2_S odour	Grey to dark brown, flowing, jelly-like with plant residues, top layers have a bit of rough sand, lower layers become denser	Predominantly yellowish-green, sometimes light brown, flowing, jelly-like, contains poorly decomposed peat admixture	Fark brown, Flowing in upper layers, getting jelly-like in deeper layers, with plant residues and well-decomposed peat impurities	Dark brown, Moderately flowing in the upper layer, the lower layer becomes extremely dense, mainly jelly-like, in deeper layers with admixture of sand and gravel
Additional data	Water transparency exceeding maximum depth (> 1.6 m); overgrown	Brown water lake	Overgrown	Very intense and regular water blooming; fish thirsting is observed in harsh winters; there was a Ludza bird integrated plant, later a meat processing plant nearby (water intake for economic needs (1000 m^3^/day)	Regular water blooming

**Table 2:**
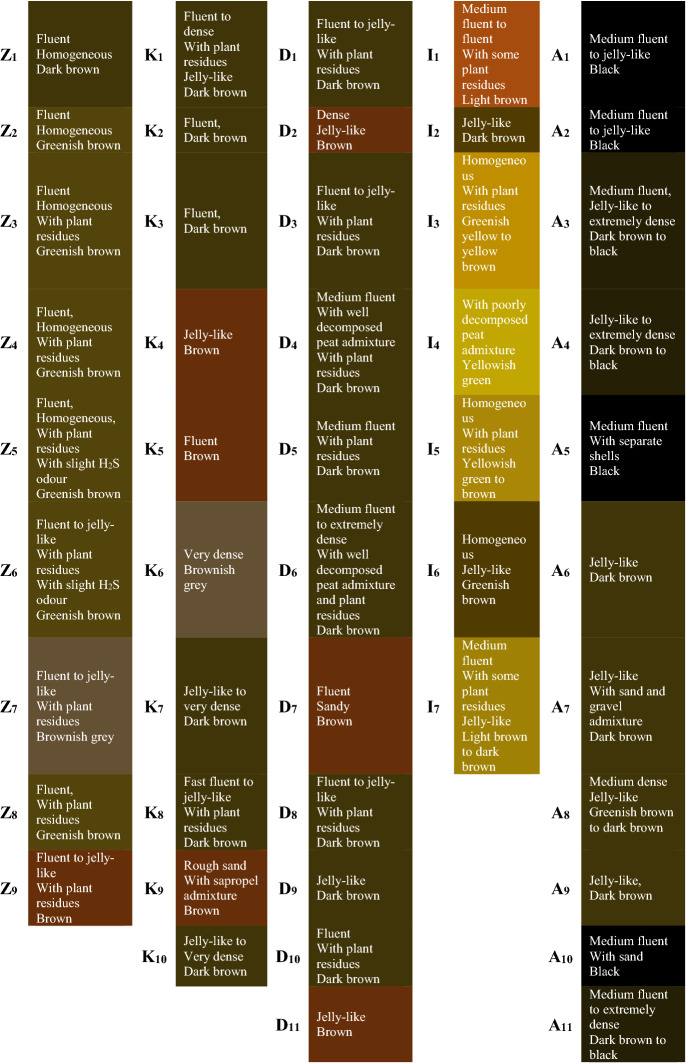
Lithological description of the sapropel exploration points.

High silica content usually relates to green and yellow colored sapropel and could be found in moraine lakes. The high organic matter relates to black colored sapropel and could be found in the lakes with low mineral content.

Sapropel consists of a sludge solution, a skeleton, and a colloidal complex. The sludge solution contains water and dissolved substances that mean mineral salts, low molecular weight organic substances, vitamins, and enzymes.

Brown and dark green sapropel affiliate the mixed type of sapropel and its origin comes from lake’s plankton, plants and sometimes connected with peat existence. This type of sapropel mostly can be found in Latvian lakes. Sapropel sample pH level is around 7–8 it means that these sapropel sediments have high mineral content (Table 3).

**Table 3:** Concentration of metals, humic and fulvic acids, total organic carbon and microbiology.

Lake↓	pH	Total concentration, mg/ml				Metal concentration, ppm							Microbiology, uncertainty ± 15,40	
		TOC	HA	FA	TPC	Pb	Cd	Ni	Co	Cu	Cr	Sb	CFU/g	Isolated species
Zeilu	7.8	126.4	160.2	74.3	77.2	2.60	0.1	11.8	5.0	9.9	20.1	0.3	2,65 × 10^6^	Serratia fonticola/Pseudomonas veronii/Pseudomonas chlororaphis
Mazais Kivdalova	7.3	129.1	167.8	72.9	103.6	2.66	0.2	18.4	8.2	12.0	27.2	0.4	2,0 × 10^5^	Pseudomonas veronii
Ivusku	8.0	106.5	113.1	76.5	70.3	3.10	0.2	3.1	1.7	3.9	9.1	0.3	1,1 × 10^5^	Paenibacillus amylolyticus/Aeromonas bestiarum
Dunakla	8.0	104.3	138.4	44.5	62.4	5.23	0.2	15.3	5.7	9.4	29.4	0.3	2,3 × 10^7^	Aeromonas sobria/Pseudomonas marginalis,/Brevundimonas diminuta
Audzelu	7.1	125.4	161.8	70.0	118.5	5.84	0.2	25.2	6.3	13.3	52.4	0.4	2,1 × 10^5^	Acinetobacter johnsonii

The following factors identified for standardizing and describing sapropel: organoleptic testing (visual look, consistency and smell, coarse composition test), test for heavy metal residue, test for pesticide residue, bacteriological test and pH^44^.

Heavy metals are one of the most widespread and significant contaminants in sediment, causing serious environmental effects due to their toxicity, persistence, and bioaccumulation.

Lake sediments can be polluted in many ways, mainly man-made pollution, such as sewage disposal, runoff from agricultural land, lakes and transport from nearby roads. However, the accumulation of heavy metals in lake sediments is not always associated with anthropogenic pollution, and the sediment may be naturally "enriched" with various metals influenced by the local geochemical background. The presence of lead and cadmium in the upper layers may indicate anthropogenic effects. In contrast, metals such as chromium, cobalt, copper and nickel are naturally occurring. Nickel can be found naturally in sediment, erosion and mineral dissolution, as well as in the catchment natural processes, as well as copper, and cobalt. The presence of nickel in the sediment may be elevated if there are municipal waste dumps in the vicinity of the mining site, while the presence of copper may indicate clayey sediment.

The determination of metals, lead (Pb), cadmium (Cd), nickel (Ni), cobalt (Co), copper (Cu), antimony (Sb) and chromium (Cr) in sediments is very important in view of their toxic effects on the environment and their long sustainability in the environment. The analysis of metal contents in the sapropel provides information on the natural and anthropogenic origin of the metal flow in the lake’s ecosystem and the influence on sapropel application in medicine. Pb, Cd, Co, Ni and Cu were present in all samples, but none of the metals exceeded maximum acceptable level compared with SCCS’s^44^ calculated values that tolerated in a different kind of cosmetic (Pb – 20 ppm, Cd – 5 ppm, Ni – 200 ppm, Co – 70 ppm, Cr(III) – 100 ppm, Sb – 100 ppm). The presence of the Pb and Cd in the upper layers of sediments indicates anthropogenic impacts growth on the lake ecosystem. In some samples, the slightly increased Ni concentrations are associated with its natural origin deposited in sediments^45^. Also in very high concentrations, Ni has low potential mobility and low ecological risk. Anthropogenic metals as Co correlated with Ni. The major route of exposure expected to be via the skin, although the potential for absorption of heavy metals through the skin is relatively low.

The use of sapropel in medicine requires that water and sapropel samples are free from pesticide residues and their contents comply with regulatory requirements.

Chlororganic pesticides are among the first to be widely used as effective help to combat unwanted plant pests and pathogens and have bioaccumulation and bioconcentration capabilities. These pesticides include persistent organic pollutants (POPs) which can move very long distances through air and water and accumulate in terrestrial and water ecosystems. The most commonly known and most widely used POP pesticide in the world is DDT.

Some water and sapropel samples at different depth levels showed the presence of DDT (dichlorodiphenyltrichloroethane) pesticide and its decomposition product DDE, DDT was a commonly used pesticide for insect control in the 20rd Century^46^. On 23th June 2015, the International Agency for Research on Cancer (IARC), a part of the World Health Organization specialized agencies of the United Nations, has evaluated the carcinogenicity of the insecticide gamma-hexachlorocyclohexane (lindane) and DDT and the herbicide 2,4-dichlorophenoxyacetic acid^47^.

Although DDT has been banned in most countries since 1970, its degradation products are very persistent and can still be found in the environment and in animal and human tissues worldwide.

Limit values for DDT concentrations contained in Legal Acts of the Republic of Latvia Nº 118 Annex 1, Table 2 “*Environmental Quality Standards for Hazardous Substances in Surface Waters*”, where the average annual concentration of DDT is 0.025 µg /L or ppb and para-para DDT – 0.01 µg /L or ppb.

Comparing lakes the concentration of DDE/DDT was slightly different. The concentrations of DDE/DDT found in surface water from lakes were in general lower than those found in samples of sapropel. The highest levels of DDE/DDT were found in all depth of Mazais Kivdalova and Zeilu and in the 2nd extraction site of Audzlu lake, but the amount of DDE / DDT was below the limit of quantification, QL.

Among the most important groups of organic acids in sapropel are humic acids (HA) and fulvic acids (FA), which are naturally resistant, high-molecular heterogeneous compounds. They consist of both aromatic structures and aliphatic circuits with different functional groups.

Humic substances make up the highest recent amount of carbon on earth that can affect the climate, soil fertility, and degradation^48^. The properties of polyphenols in humus substances in cosmetics and medicine can be used as antioxidants. Antioxidant (AO) activity was determined in fulvic acid with a concentration of carbon fraction (FA-C) till 700 mg/L. AO activity has been measured by a various methods such as Total phenolic concentration (TPC), Total Antioxidant Status (TAS) level, 2,2′-azino-bis(3-ethylbenzothiazoline-6- sulphonic acid (ABTS), and DPPH radical scavenging assays. Results revealed that the AO activity is dependent on the concentration of carbon fraction in FA because lower concentration was not sufficient to scavenge free radicals due to its low TAS level. One tendency is that one of the lakes – Dunaklu – gives considerably lower both AO and humic and fulvic acids levels. However, Ivusku Lake with the lowest AO levels is high at the FA level. The concentration of humic acid and FA and the AO levels varies strongly between different lakes. It was found that AO level is considerably higher in organic sapropel extracts from the lakes Audzelu, Mazais Kivdalovas, and Zeilu. The total AO level is almost threshold between the highest and lowest values. The difference in HA (max. 167.8 mg/ml, min. 113.1 mg/ml) levels between different lakes much or less pronounced than the difference in the FA (max. 76.1 mg/ml, min. 44.5 mg/ml) and AO levels (Table 3).

Therapeutic mud has bactericidal and bacteriostatic (antimicrobial) properties. A special role belongs to the microflora contained in peloids, on the vital activity of which the biological processes occurring in them depend. There is a huge amount of microorganisms in the healing mud – billions per 1 g of peloid. They take part in the breakdown of organic substances, which are closely associated with the formation of mud and the regeneration of spent therapeutic mud.

By oxidizing the organic matter formed at the bottom of the reservoir, with the help of oxygen taken from sulfates – salts of sulfuric acid, seawater, microorganisms get the energy necessary for life.

The high microbiological activity of peloids is their characteristic feature that distinguishes peloids from other similar formations. The active activity of bacteria, fungi, other components contributes to the decomposition of organic and animal residues and enriches therapeutic mud with humic substances, bitumen, produces hydrogen sulfide, ammonia, carbon dioxide, and other gases; only the constant activity of microbes ensures a stable content in the mud of such unstable microcomponents as vitamins, enzymes, and hormones. Due to the microorganisms present in the mud, they are able to self-clean after anthropogenic pollution in the deposits and regenerate after use in the mud baths.

Sapropel is still a living material with its specific biome and microbiological flora that not identified yet in detail. Totally, the nine species of bacteria found in the lakes. The Dunaklu Lake has the highest value of CFU/g – 2.3·10^7^, three species of bacteria were most prevalent and one of them – *Aeromonas sobria* – is an opportunistic pathogen^49^. Ivusku Lake has lowest value and two species – *Paenibacillus amyloyticus* and *Aeromonas bestiarum* – were most abundant. Both species not known as pathogens or opportunistic pathogens; however, it reported that *Paenibacillus amylolyticus* might have antimicrobial properties by producing antibiotics^50^ that could explain lower value of CFU/g in Ivusku Lake. Sapropel from Zeilu Lake contains 2.65·10^6^ CFU/g and three bacterial species are most prevalent, of those two are *Pseudomonas veronii* and *Pseudomonas chlororaphis* that are known as normal soil bacteria, and *Pseudomonas chlororaphis* may have antimicrobial properties due to production of rhamnolipids and some substances with antibiotic characteristics^51^. However, *Serratia fonticola* is an enterobacteria and opportunistic pathogen that could indicate some kind of pollution of wastewaters in this lake. Sapropel form this lake should definitely sterilized as *Serratia fonticola* known to cause skin and soft tissue infections.

Sapropel form Audzelu Lake and Mazais Kivdalova Lake have similar values of CFU/g and have single dominant species – *Acinetobacter johnsonii* (emerging as a fish pathogen) in Audzelu Lake samples and *Pseudomonas veronii* in Mazais Kivdalova Lake.

The Scientific Commission on Consumer Safety has developed guidelines for the use of various mineral, animal and plant-based and biotechnological ingredients in cosmetic products^52^. These guidelines may apply to sapropel as the topical application to the skin or in the form of various gels, creams, shampoos and other products for external use. EU guidelines state that cosmetic products must not contain microbial pathogens and the total aerobic microorganisms must be low. For cosmetic products intended for paediatric or use near to eye zone (Category 1), the CFU or colony-forming units shall according to EU Regulation should not exceed 100 CFU/g (bacteria, yeasts, fungi) and for other cosmetic products (Category 2) should not exceed 1000 CFU/g. Other specific microorganisms such as *Escherichia coli*, *Pseudomonas aeruginosa*, *Staphylococcus aureus*, *Candida albicans*, and others presence should not be observed in 1 g or 1 ml in both Category 1 and 2 end products. Consequently, sapropel, which is intended to be used in various topical applications, also requires routine tests for microbiological and contaminant monitoring. Adaptation of ISO Standards recommended for quality routine tests and use – ISO/TR 19838: 2016, ISO 21148: 2017, ISO 17516: 2014. For the detection of *Aerobic Mesophyll Bacteria* – ISO 21149: 2017, *Staphylococcus aureus* – ISO 22718: 2015, *Pseudomonas aeruginosa* – nd yeast—ISO 16212: 2017 standards needed^53^. It is important to evaluate the utilization of sapropel material after use in therapeutic or cosmetic applications to prevent organic and microbial contamination of the environment. One of the methods, if the material used is of low toxicity or non-toxic and not in high volume, is to dilute it with large amounts of water and then ensure that it is discharged into the sewage system, prevent entry into natural waterways^54^. If preservatives or other compounds are added to the material used before or after application, it should be considered their occurrence in nature, effects on flora, fauna, and potential degradation time.

## Conclusions

The use of organic-rich lake sediment like sapropel considered a solution because of the necessity for wider use of the local resources and available natural materials and due to previously insufficient research of sapropels for pharmaceutical needs.

The appropriate sapropel layer found from 2.0 to 9.0 m (actual layer – 0.9–11.4 m) from the surface of the sediment layer. If the depth is less than 1.5 m from the surface of the sediment layer, sapropel sediments are not fully developed.

Sapropel is a jelly-like homogeneous mass, its texture in upper layers is close to cream-like, and in lower layers, it becomes denser. The sediments are odorless except the separate types that smell of H_2_S. Organoleptically sapropel found from greenish-yellow (with high silica content) to almost black (with high organic matter and low mineral content). Brown and dark green sapropel, mostly found in Latvian lakes affiliate to the mixed type of sapropel and comes from lake’s plankton, plants and sometimes connected with peat existence. Sapropel pH level is around 7–8, it means that sediments have high mineral content.

Pb, Cd, Co, Ni, and Cu were present in all samples, but none of the metals exceeded the maximum acceptable level compared with SCCS’s calculated values. The presence of the Pb and Cd in the upper layers of sediments indicate anthropogenic impacts growth on the lake ecosystem. In some samples, the slightly increased Ni concentrations are associated with its natural origin deposited in sediments. Also in very high concentrations, Ni has low potential mobility and low ecological risk. The major route of exposure expected to be via the skin, although the potential for absorption of heavy metals through the skin is relatively low.

Some water and sapropel at different depth levels showed the presence of DDT pesticide and its decomposition product DDE. The concentrations of DDE/DDT found in surface water from lakes were in general lower than those found in sapropel. DDE/DDT was found in all depth of Mazais Kivdalova and Zeilu and in the 2nd extraction site of Audzlu lake and the amount was below the limit of quantification.

The total AO level is almost threshold between the highest and the lowest values. The difference in HA (max. 167.8 mg/ml, min. 113.1 mg/ml) levels between different lakes much or less pronounced than the difference in the FA (max. 76.1 mg/ml, min. 44.5 mg/ml) and AO levels.

Despite the fact that in raw sapropel samples no active pathogens identified, CFU exceeds the limit allowed by tenfold or more in all of sapropel samples. It is necessary to reduce CFU/g in the raw sapropel by sterilization or by adding preservatives before using it in cosmetic or medical applications.

In the framework of this study, the Guidelines for the extraction of sapropel have been developed, thus, practically all the activities that the sapropel industrial miners would have to perform.

## Supplementary information

Supplementary information.

Supplementary information.
