# Supplementary figures and images for: Assessment of sapropel use for pharmaceutical products according to legislation, pollution parameters, and concentration of biologically active substances
Source: Sci Rep. 2020 Dec 9;10:21527. doi: 10.1038/s41598-020-78498-6 (PMC7725781; doi:10.1038/s41598-020-78498-6)

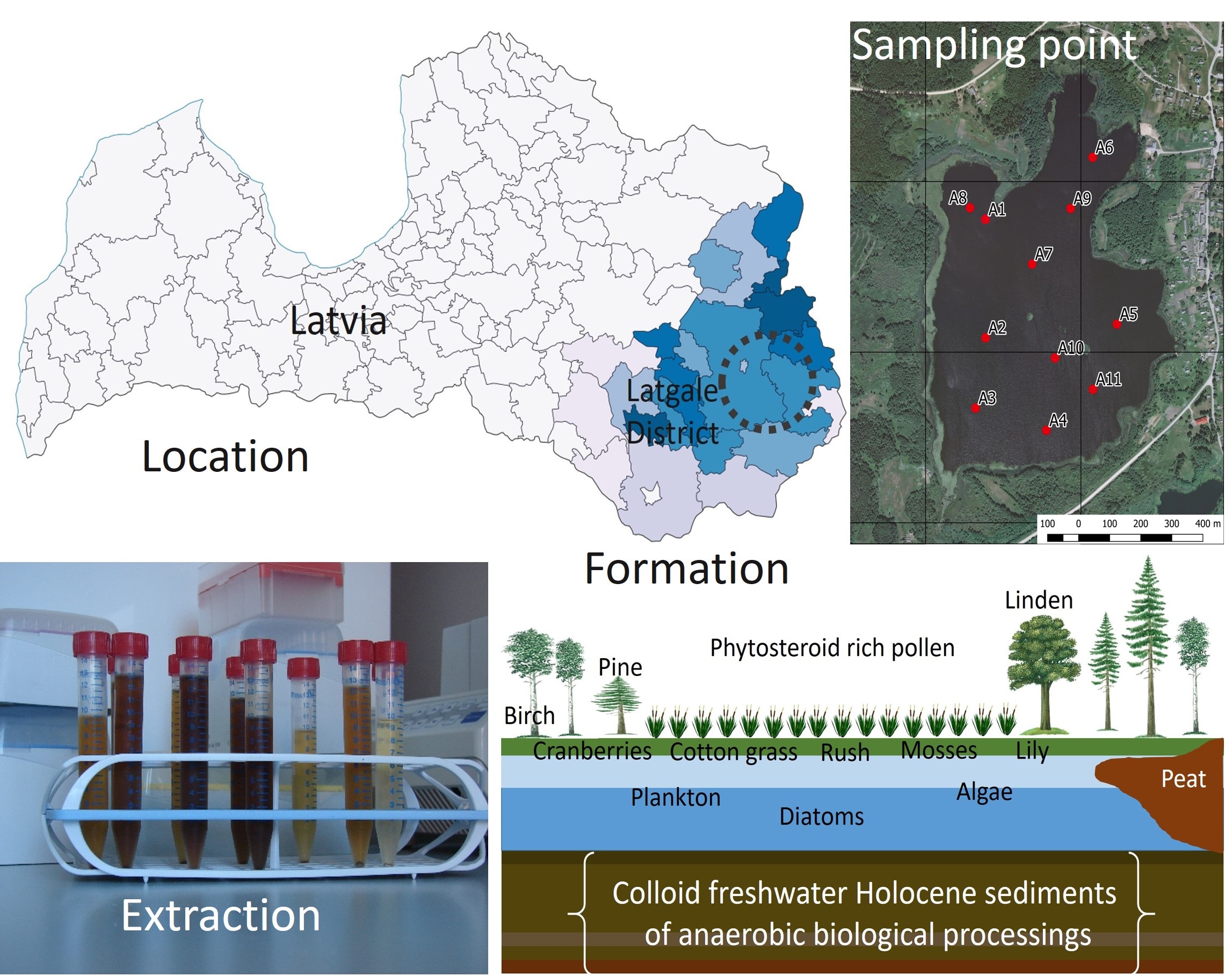

Supplement: Supplementary file 1 — Supplementary information. [file 41598_2020_78498_MOESM1_ESM.jpg]

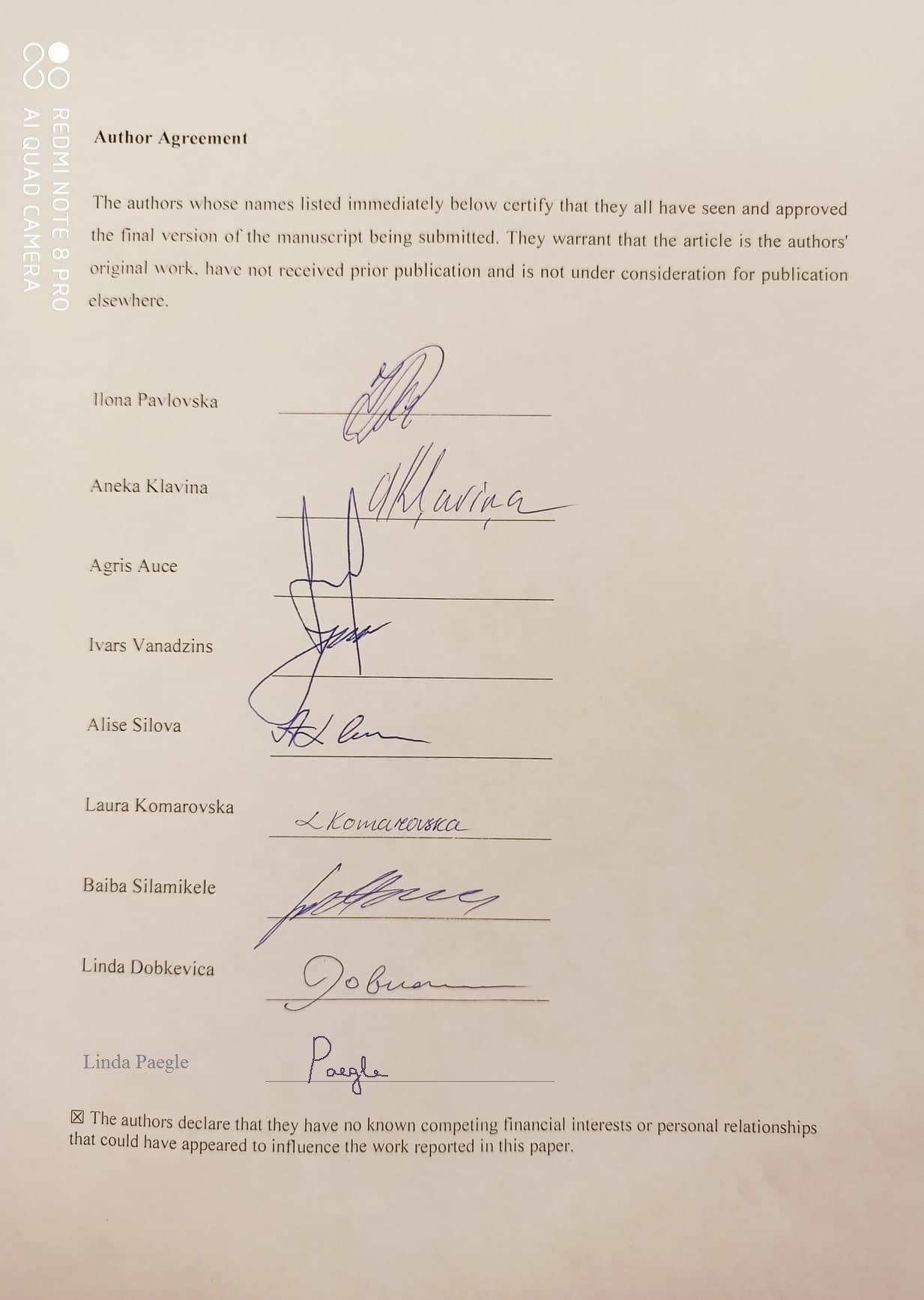

Supplement: Supplementary file 2 — Supplementary information. [file 41598_2020_78498_MOESM2_ESM.jpg]
